# A Multicentric Observational Study to Determine Myocardial Injury in Severe Community-Acquired Pneumonia (sCAP)

**DOI:** 10.3390/antibiotics12121710

**Published:** 2023-12-08

**Authors:** Ignacio Martin-Loeches, Giampaolo Maggi, Emili Diaz, Judith Marín-Corral, Alfonso Guedea, Marcos I. Restrepo, Luis F. Reyes, Alejandro Rodríguez

**Affiliations:** 1 Department of Intensive Care Medicine, Multidisciplinary Intensive Care Research Organization (MICRO), St James’s Hospital, D08NYH1 Dublin, Leinster, Ireland; giampaolo.maggi@gmail.com; 2 Pulmonary Intensive Care Unit, Respiratory Institute, Hospital Clinic of Barcelona, IDIBAPS (Institut d’Investigacions Biomèdiques August Pi i Sunyer), University of Barcelona, CIBERes, 08080 Barcelona, Spain; 3 Department of Intensive Care, Emergency Medicine and Anesthesia, Fondazione Policlinico Universitario A. Gemelli IRCCS, 00168 Rome, Italy; 4 Critical Care Department—Hospital Parc Tauli/UAB, 08208 Sabadell, Spain; emilio.diaz.santos@gmail.com; 5 Critical Care Department—Hospital del Mar, 08003 Barcelona, Spain; jmarincorral@gmail.com; 6 Radiology Department—Hospital Universitari de Tarragona Joan XXIII, 43005 Tarragona, Spain; alfonsogudea@jviii.es; 7 Medicine Department—South Texas Veterans Health Care System, University of Texas Health, San Antonio, TX 77030, USA; restrepom@uthscsa.edu; 8 Unisabana Center for Translational Science, School of Medicine, Universidad de La Sabana, Chia 250001, Colombia; luis.reyes5@unisabana.edu.co; 9 Clinica Universidad de La Sabana, Chia 140013, Colombia; 10 Pandemic Sciences Institute, University of Oxford, Oxford OX1 3BD, UK; 11 Critical Care Department—Hospital Universitari de Tarragona Joan XXIII/URV/IISPV/CIBERES, 43005 Tarragona, Spain; ahr1161@yahoo.es

**Keywords:** sCAP, sepsis, septic shock, ICU, Pneumonia, MOF, endothelial damage, MRI, S pneumoniae

## Abstract

**Background:** Severe community-acquired pneumonia (sCAP) is the most frequent admission for acute respiratory failure in intensive care medicine. Observational studies have found a correlation between patients who were admitted with CAP and the development of cardiovascular events. The risk of acute myocardial damage in patients with CAP is particularly high within the first 30 days of hospitalization. **Research design and methods:** Multicenter prospective cohort analysis conducted in consecutive patients admitted to an ICU with microbiologically confirmed diagnoses of sCAP. The aim was to determine any structural cardiac damage detected by advanced imagining techniques (cardiac MRI) and cardiac biomarkers in patients with sCAP. The patients were stratified, according to their etiology, into pneumococcal or not-pneumococcal sCAP. The primary outcome was cardiac damage at day 5 and 7 of clinical presentation. **Results:** A total of 23 patients were consecutively and prospectively enrolled for two winter periods. No significant differences were observed between the median troponin when comparing the pneumococcal vs. non-pneumococcal. The incidence of myocardial damage was numerically higher in the pneumococcal subgroup (70% vs. 50%, *p* = 0.61) on day 5 and on day 7 (53% vs. 40%, *p* = 0.81) but did not achieve significance. Confirming a correlation between the biomarkers of cell damage and the biomarkers of myocardial damage, only a positive and significant correlation was observed between h-FABP and DNA on day 1 (r = 0.74; *p* < 0.01) and day 3 (r = 0.83; *p* < 0.010). Twenty cardiac MRIs were performed on the 23 patients (87%). No presence of fibrosis was observed in any of the studies carried out within the first 15 days of admission. **Conclusions:** No significant myocardial damage was found in patients with sCAP independent of the bacterial etiology in accordance with biomarker alterations (Troponin and/or h-FABP) or cardiac MRI. Using cardiac MRI, we could not find any presence of myocardial fibrosis within the first 15 days of admission.

## 1. Introduction

Lower respiratory tract infections (including CAP) are the fourth most common cause of death worldwide. Severe community-acquired pneumonia is the most frequent admission for acute respiratory failure in intensive care medicine (ICU) [1]. Patients who need invasive mechanical ventilation and/or vasopressors have a high risk of in-ICU or in-hospital mortality [2,3,4]. Over the last years, some studies have found a correlation between patients who were admitted with CAP and the development of cardiovascular events [5]. The risk of acute myocardial damage in patients with CAP is particularly high within the first 30 days of hospitalization [6].

Recently, animal models with sCAP suggest the development of myocardial damage and cardiac injury, including cardiac scarring, via apoptosis, and other cellular damage mechanisms, such a cardiomyocyte death and collagen deposition after antibiotic treatment [7]. It is yet unknown whether these findings also occur in sCAP patients, manifested by major adverse cardiovascular events (MACE) in the form of direct cardiac cytotoxicity (e.g., cell death), heart failure, clinically relevant arrhythmias, or acute coronary syndrome [8]. Pneumococcal pneumonia can develop cause MACE in one out of three patients and is more frequent in those with cardiovascular comorbid conditions. It is also being shown in animal models that S. pneumoniae carries virulence factors, such as pneumolysin [9], that can induce cardiomyocyte cell wall damage with subsequent necroptosis (a programmed form of necrosis or inflammatory cell death) in the lungs (pulmonary macrophages) of patients with CAP [2,3,10].

In this manuscript, we performed exploratory, pilot, observational analyses, conducted on consecutive patients admitted to the ICU with microbiologically confirmed diagnoses of sCAP, with the hypothesis that patients with sCAP develop structural cardiac damage that can be detected by advance imagining techniques and with biomarker alterations. The main objective was to detect the presence of myocardial damage using magnetic resonance imaging (MRI) and to analyze myocardial damage biomarkers and the correlation of these with the degree of cell damage, considering the severity of illness in patients with sCAP.

## 2. Material and Methods

### 2.1. Study Design

This was a multicenter prospective cohort analysis (ClinicalTrials.gov Identifier: NCT03058211) (accessed on 1 July 2021) [11]. The baseline characteristics, clinical outcomes, and prognostic factors related to myocardial damage were assessed. This study was approved by our institutional review board (Institutional Review Board of Pere Virgili Health Institute-IISPV, number 5037/03/2017) and in each participating institution, and each patient signed informed consent to be included in the study.

### 2.2. Subjects

We prospectively enrolled patients with microbiologically confirmed sCAP who were treated in the three participating ICUs for two winters period sessions in 2017–2019.

Inclusion criteria were (1) Patients with microbiologically confirmed severe community-acquired pneumonia (sCAP) and the need for either invasive mechanical ventilation and/or vasopressors due to the presence of sepsis at the onset [3]; (2) Over 18 years old and under 65 years old; (3) No history of ischemic heart disease; 4) Informed consent signed. Exclusion criteria were (1) Over 18 years or under 65 years; (2) No IC signature; (3) Healthcare-associated pneumonia (HCAP) or nosocomial pneumonia; (4) Viral pneumonia or viral co-infection; (5) History of ischemic heart disease, myocarditis, primary arrhythmias, and/or drugs that might cause or lead to arrhythmias; (6) Statin medication; (7) Patients with transplantation, primary lung tumor, or advanced tumors at other sites; (8) Presence of leukopenia or neutropenia (unless due to pneumonia); (9) Adjuvant therapy for severe immunosuppression in human immunodeficiency virus-positive (HIV) patients (CD4 < 100); (10) Patients with previous underlying pulmonary diseases (e.g., COPD, asthma, etc.) requiring long-term home oxygen therapy. 

### 2.3. Data Collection and Definition

Demographics: Clinical data was collected in a detailed database from patients admitted to ICU using an electronic medical record system (AMR). Our data were recorded by attending nurses and doctors when patients were admitted to the ICU. The demographic characteristics of each patient, including comorbidities, were reviewed thoroughly. We collected the patients’ worst vital signs, laboratory results, ventilator support, and use of pressors within 24 h of admission before initiation of ICU treatment. Duration of fever, infection markers, respiratory management, and antibiotics were recorded during ICU treatment.

Microbiological diagnosis: All patients had a sample of sputum or an endotracheal aspirate if intubated. Microbiological diagnosis required a positive result from respiratory tract samples that underwent Gram staining and quantitative culture. Urine antigen testing was used to screen for *Streptococcus pneumoniae* and *Legionella pneumophila* and immunoglobulin antibody testing screened for *Chlamydia pneumoniae* and *Mycoplasma pneumoniae.* Severity was assessed by the acute physiology and chronic health evaluation (APACHE) II score, sequential organ failure assessment (SOFA), confusion, uremia, respiratory rate, BP, and age ≥ 65 years (CURB-65), and clinical pulmonary infection score (CPIS).

### 2.4. Biomarker Analysis

#### 2.4.1. Cardiac Damage Biomarkers

Collection of blood serum biomarkers was carried out during the hospitalization on days 1, 3, and 7. An ultrasensitive Troponin T (Tn) value greater than the 99th percentile of the normal reference population was established as a diagnostic criterion for myocardial damage (MyD), which constituted the upper reference limit; in our case, this was 47 ng/mL [2]. N-terminal pro-brain natriuretic peptide (NT-proBNP) with a cut-off point of >900 pg /mL was considered for the diagnosis of highly probable heart failure, according to the limits of our laboratory and related to ages between 50 and 75 years. NT-proBNP was determined because it is a more stable and longer-lasting molecule than BNP. The determination was performed using the KIT Human NT-ProBNP ELISA Kit (Sandwich ELISA), Company LifeSpan BioSciences (Lynnwood, WA, USA).

Heart fatty acid binding protein (H-FABP) is the major isoform found in the heart, highly specific for the myocardium, 15–20 times more specific than myoglobin, and it is released extremely quickly from the ischemic myocardium [3,4]. According to the literature, myocardial damage is defined when the H-FABP cut-off point is >7 ng/mL [4]. H-FABP was measured by KIT DY1678 Human h-FABP ELISA (R&D system according to the manufacturer’s technique).

#### 2.4.2. Cell Damage Biomarkers

Circulating histones: The determination was performed by QUANTA Lite^®^ Histone Kit (Werfen Inc, Barcelona, Spain), an assay based on the ELISA (enzyme-linked immunosorbent assay) technique for the semi-quantitative detection of anti-histone antibodies in human serum, according to the manufacturer’s instructions. Measurements were performed on days 1, 3, 5, and 7. Values higher than 0.1ug/ mL were considered abnormal and indicative of cell damage.

Neutrophil extracellular traps (NETs): Quantification of NETs was performed by using the methodology based on the green fluorescent dye (Quant-ITTM PicoGreenR dsDNA) that binds to DNA. The fluorescence intensity (emission at 485-nm wavelength) is a measure of the fluorescence intensity (emission at 485-nm wavelength) by the amount of DNA and quantified by a reader (fluorimeter). The extreme cut-off points of the method are 50/mL to 3000 ng/mL. A calibration curve with calf thymus DNA was provided in the method kits.

Interleukin-1 was determined by Elisa analysis (Procarta Cytokine Inmunoassay Kit) of Affymetrix (Rafer).

### 2.5. Cardiac Imaging

Cardiac magnetic resonance imaging (cMRI) is a suitable noninvasive method for adequate and early recognition and quantification of cardiac fibrosis (scarring) through the retention of the contrast agent (gadolinium) in the myocardial tissue. cMRI was performed using a protocol agreed upon by the participating centers, as published by Ambale-Venkatesh and Lima [12], consisting of (a) late gadolinium enhancement—a technique that can be used to identify the presence, pattern, and size of replacement or focal fibrosis, and has proven prognostic capacity—and (b) T1 mapping—a technique that allows for the accurate quantitation of diffuse and infiltrative interstitial fibrosis and has tremendous prognostic potential in a wide variety of ischemic and nonischemic diseases. cMRI was performed between days 7 and 14, when cardiac fibrosis lesions secondary to bacterial myocardial invasion were expected to occur. If the result was positive, a second MRI was performed between 6 and 8 months after discharge (convalescence period) to assess whether the observed alterations persisted, new lesions emerged, or the was resolution of previously identified lesions.

### 2.6. Objectives

The primary objective was to determine the incidence of myocardial damage by cardiac cMRI in patients with sCAP. Secondary objectives were (1) To determine the presence of myocardial scarring by a non-invasive method (cardiac MRI); (2) To evaluate whether myocardial damage is related to the etiology sCAP (pneumococcal vs. non-pneumococcal); and (3) To identify myocardial damage predictive factors to be able to stratify patients according to the risk of suffering cardiovascular events to try to prevent complications and mortality.

### 2.7. Statistical Analysis

Kolmogorov–Smirnov test was used to test the normality of continuous variables. The continuous variables conforming to the normal distribution were compared using the independent sample T-test and were expressed as mean (SD). All continuous variables not conforming to normality were expressed as median (IQR) and were compared by Mann–Whitney test. The categorical variables were expressed as frequency and percentage and were compared using the probability ratio χ^2^ test. Sample size calculation: According to published data [8], cardiovascular events occur in 17% of patients with severe CAP. We expected to find, by cardiac magnetic resonance imaging, 10% myocardial scarring in patients with pneumococcal CAP vs. 0% in patients with non-pneumococcal CAP. For a confidence level of 95% and a power of 90%, the number of patients that needed to be included was 21 (Supplementary Material Figure S1). Statistical analysis and graphic rendering were performed using SPSS26.0 and GraphPad Prism 9.0. Double-tailed *p* < 0.05 was considered statistically significant.

## 3. Results

### 3.1. Population Description

A total of 23 patients were consecutively and prospectively enrolled. Patients were divided into pneumococcal or non-pneumococcal sCAP. Patients with sCAP showed a higher severity of CURB-65, shorter antibiotic administration, and a higher need of HFOT but similar clinical outcomes. Further demographic and clinical characteristics are listed in Table 1.

### 3.2. Cardiac Biomarkers

The median level of Tn (ng/L) on admission was 19.3 (14–27), and this increased significantly on day 3 (214) and decreased on day 7, though the values remained higher than those observed on admission (39). According to the cut-off point established for myocardial damage, only one patient met the myocardial damage criteria on admission. However, on day 3, 61% (n = 14) presented with myocardial damage, and this persisted in almost half (48%) of the patients on day 7. No significant differences were observed between the median Tn when comparing the pneumococcal vs. non-pneumococcal subgroups. The incidence of myocardial damage was higher in pneumococcal (70% vs. 50%, *p* = 0.61) on day 5 and on day 7 (53% vs. 40%, *p* = 0.81) but did not achieve significance.

No differences were observed in h-FABP levels between the pneumococcal vs. non-pneumococcal subgroups. The presence of myocardial damage defined by an h-FABP was considerably lower than that observed with Tn. Only two patients (8.7%) met the criteria for myocardial damage based on h-FABP on admission to the ICU, and only 4% remained with h-FABP above the cut-off point at 3 and 7 days. The frequency of the presentation was higher in the non-pneumococcal subgroup.

We found NT-proBNP levels higher than 900 pg /mL in most of the patients. No significant differences were observed in NT-proBNP levels or in the incidence of myocardial damage among the different patient groups during the observation period.

In Table 2, cardiac biomarker (Tn, h-FABP, and NT-proBNP) levels are displayed in accordance with the defined time points. There was a negative correlation (Pearson) r = −0.72 and significant (*p* < 0.001) between Troponin and NTpro-BNP on day 3.

### 3.3. Inflammatory Biomarkers

**Figure 1:**
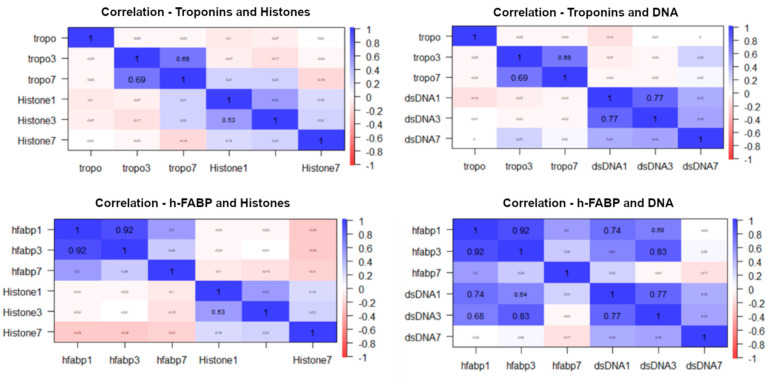
Correlation for biomarkers of cell damage and myocardial damage at days 1 and 3.

Elevated levels of IL-1 were observed on admission, with significant decreases until day 7. It was similarly observed in the pneumococcal and non-pneumococcal subgroups, although, in the latter subgroup, the decrease did not achieve significance, possibly due to a type 1 error. No significant differences were observed between the subgroups. A significant increase in circulating histones was observed from admission to day 7. This trend was similar in both subgroups, but in the non-pneumococcal subgroup, this increase did not reach significance. No significant differences were observed between subgroups, although, on day 7, the difference was borderline significant. A significant reduction in circulating DNA levels was observed from admission to day 7 in the overall population and subgroups, with no differences between the subgroups regarding the days of observation (Table 3). When performing the correlations between the biomarkers of cell damage (histones and DNA) and the biomarkers of acute myocardial damage (Tn and h-FABP), only positive and significant correlations were observed between h-FABP and DNA on day 1 (r = 0.74; *p* < 0.01) and day 3 (r = 0.83; *p* < 0.01) (Figure 1).

### 3.4. Cardiac MRI

A total of 20 cardiac MRIs were performed on the 23 patients (87%). In the remaining 3 patients, the MRI could not be completed due to claustrophobia syndrome. No presence of fibrosis was observed in any of the studies carried out within the first 15 days following admission. It should be noted that in the two patients who died, who belonged to the pneumococcal subgroup and had bacteremia, an autopsy was performed, which also did not show the presence of acute myocardial fibrosis.

### 3.5. Clinical Outcomes and Myocardial Damage

**Figure 2:**
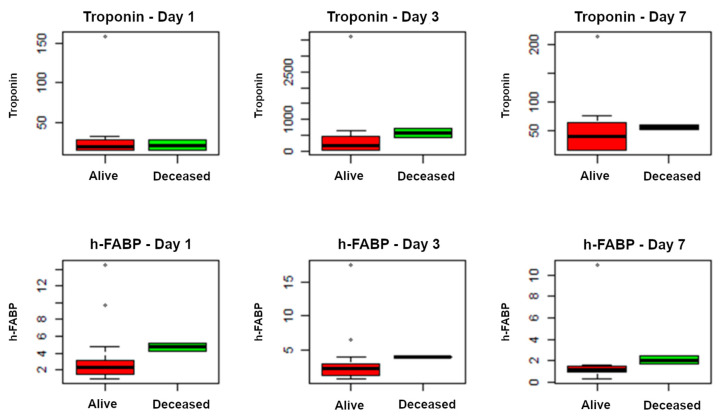
Correlation between biomarkers of myocardial damage (Troponin and h-FABP) and mortality at day 1, day 3, and day 7.

The crude overall mortality in the ICU was 8.7% (n = 2/23), and it was non-significantly higher in the pneumococcal subgroup (15.4%, n = 2/13) compared to the non-pneumococcal (0%, *p* = 0.5) subgroup. Although higher values were observed in the deceased compared to the survivors, only h-FABP on day 7 was significantly higher in the deceased (*p* = 0.04), as is shown in Figure 2 and Table 4.

## 4. Discussion

The present manuscript aimed to determine the parallelism of findings in animal models with sCAP by identifying structural myocardial damage in patients with CAP. We consider cardiac MRI as the best tool to detect cardiac scarring because of myocardial damage, but we were unable to find any myocardial severe injuries [13,14]. We also measure several biomarkers, intending to determine some degree of myocardial damage and to characterize and potentially match with the degree of cell damage in the patients. Again, we were unable to demonstrate such an association.

When a patient is admitted to ICU with sCAP, the chances of survival could be as low as 50% in cases where the patient needs to be under invasive mechanical ventilation and requires vasopressor agents [2]. Apart from this short-term mortality, which usually occurs within the first 30 to 60 days after ICU admission, many reports show potential MACE development within the hospital stay after the patient is discharged from the ICU [4]. It has also been previously reported that some high coagulability can occur in some patients with sCAP, such as those with COVID-19 pneumonia, which may cause cardiac damage [15]. More recently, many research groups have reported a delay in the presentation of cardiovascular events, hypothesizing that this is because of a prolonged but low degree of cell damage [8]. In our manuscript, we aimed to determine anatomical injuries that can at least partially explain those late-onset complications. We could not do so using a state-of-the-art imaging technique with cardiac MRI. This process was extremely challenging since some patients were not fit enough to undergo a procedure that needed quite some time. Luckily, we could perform up to 90% of cardiac MRIs, which allowed us to be confident that our results are not biased due to a high number of missing data.

We performed a multidimensional approach, not only using imaging but also assessing cardiac damage at a molecular level and using cardiac biomarkers. We found elevated troponin at day 3 in two out of three cases; however, H-FABP was extremely low (4%). When analyzing published pooled data, a metanalysis that included 25 studies reporting the incidence of cardiac events within 30 days of pneumonia diagnosis reported cumulative rates of new or worsening heart failure (14%, range 7–33%), new or worsening arrhythmias (5%, range 1–11%), and the acute coronary syndromes myocardial infarction or unstable angina (5%, range 1–11%) in patients admitted to the hospital with pneumonia (7). There is a big gap between the two chosen biomarkers (Tn; h-FABP) for detecting myocardial damage and the literature incidences. This discrepancy when comparing our data and pooled data could be explain due to the heterogeneity of the type of patients, including risk factors, age, and, mainly, severity (i.e., septic shock, invasive mechanical ventilation, multiorgan failure, etc.). Another important area of uncertainty was the inability of a correlation between Troponin and H-FABP to detect myocardial damage. Both cardiac markers have been proposed in diagnosis and prognosis of acute coronary syndrome (ACS), and the better performance in ACS is when their measurement is performed together on admission. It is also relevant to say that, in the study published by Reyes et al., biomarkers, unlike invasive methods, were unable to detect the myocardial damage. This finding could suggest that while biomarkers can be very useful for myocardial damage, they are somehow not sensitive enough to correlate with tissular microscopical damage, as seen in heart biopsies.

Our study is unique in its design as we were able to perform non-invasive state-of-the art cardiac imaging by cardiac MRI. Cardiac MRI has become a very useful tool to demonstrate myocardial injury, and it is becoming widely implemented in clinical practice when non-specific myocardial injuries are suspected [16]. In our study, we relied on this technique because it is not invasive; however, it had some technical difficulties acquiring high-quality images when performed on a critically. In our study, cardiac MRI did not demonstrate the presence of any myocardial scarring in the first 15 days after admission, which could be related to the poor sensibility of MRI, which makes it difficult to detect the small collagen depositions that could be easily identified in animal models. In other words, the presence of cardiac fibrosis TGF-β in the animal heart that stimulated myofibroblasts to produce collagen to replace dead cardiomyocytes might not be detected by current cardiac MRI.

Our results also indicated that we might have had some confounding factors. In patients with sepsis and especially septic shock, there are cases where patients can show elevated Troponin levels that are not totally explained by the presence of a cardiac damage in the context of sepsis. In our cohort, bacteremia occurred in nine patients in the pneumococcal subgroup and only in one patient in the non-pneumococcal subgroup. Our study hypothesis was obtained from an animal model and all effects and interventions were extremally controlled and recorded. In our cohort, some patients received corticosteroids (one out of five patients on admission), and, in the animal study, no animals received steroids as part of the management. The administration of corticosteroids to patients with sCAP has been a matter of debate, and the consensus is that their use could be justified in the event of sCAP with the presence of septic shock. On one hand, this is associated with an easier weaning of inotropes, but does the administration of steroids reduce the inflammatory myocardial damage? Would this explain the lower degree of abnormal myocardial findings [17]?

A major finding based on our observational study was that we were unable to demonstrate any correlation between myocardial damage and pneumonia etiology. Indeed, the incidence of some myocardial injury was detected with higher frequency in the patients with pneumococcal sCAP (70% vs. 50%) but without reaching statistical significance. Additionally, we must consider that there would have been possible risk factors identified in previous studies, including older age, pre-existing congestive heart failure, and grade severity of pneumonia [18]. The small number of patients prevented us from obtaining predictive factors that would enable us to stratify patients according to the risk of cardiovascular events, to try to prevent complications. It is also important to consider that other potential etiologies might behave differently to cause myocardial damage and specific tropism for the myocardium. SARS-CoV-2 has been proven to be able to damage the myocardium by direct invasion and through multiple indirect mechanisms sustained by cell damage, but no conclusive studies have been published [19].

We must acknowledge some limitations in this study. First and foremost, the sample size needed to be increased; this could have made the study underpowered, limiting the ability to detect minor differences between groups. This might be related to a type I error, when the effect of an intervention was deemed significant when, in fact, there was no real difference or effect due to the potential infection damage [20]. In statistical terms, due to the limited sample size, we are still determining if the null hypothesis was correctly rejected, and this causes a false-positive result that could have been detected. However, this limited number of patients was in response to the strict exclusion criteria required to obtain a homogeneous population without previous cardiovascular risk factors, with the aim of trying to determine the true risk of cardiovascular events in patients with no history of this disease. Second, previous studies have focused on a much older population (the median age in our cohort was 45 years), and this could raise two potential questions: Is aging a risk factor for severe pneumonia, and is aging a risk factor for cardiovascular events [6]?

## 5. Conclusions

Even though this study has a limited sample size, making it difficult to draw solid conclusions, it is, to the best of our knowledge, the first one to determine with MRI and cardiac biomarkers myocardial damage in patients with sCAP. No significant myocadiac damage was found in patients with sCAP independent of the bacterial etiology by biomarker alterations (Troponin and/or h-FABP) or cardiac MRI. Using cardiac MRI, we could not find any presence of myocardial fibrosis within the first 15 days after admission. There was only a positive and significant correlation between h-FABP and DNA on day 1 and day 3 that could determine a link between myocardial damage and patients’ inflammatory status. This human study does not support the animal model hypothesis previously published, suggesting that cardiac scarring occurs only in non-human primate models. Despite of the small sample size, the lack of any identification of myocardial fibrosis with cardiac MRI suggests that myocardial scarring does not warrant further investigation in patients with sCAP. Further mechanisms should be adequately studied to determine the etiology of MACE.

## Figures and Tables

**Table 1:** General characteristics of the population sCAP.

	Overall (n = 23)	Pn (n = 13)	No Pn (n = 10) *	*p*
*Demographics*				
Age, median (IQR)	45 (40–50)	48 (43–51)	42 (40–50)	0.43
Sex male, n (%)	11 (47)	6 (46.1)	5 (50)	1.00
APACHE II score, median (IQR)	13 (7–17)	13 (9–15)	16 (11–17)	0.15
CURB-65, median (IQR)	2 (1–3)	**1 (1–2)**	**2 (2–3)**	**0.03**
CPIS, median (IQR)	6 (4–7)	6 (4–7)	5.5 (4–6)	0.90
SOFA, median (IQR)	4 (3–6)	3 (3–6)	5 (4–6)	0.16
Symptoms onset (days), median (IQR)	4 (2–5)	4 (2–4)	5 (2–7)	0.20
*Treatment received*				
Hours to ATB, median (IQR)	3 (1.5–4.0)	2 (2–4)	3.5 (2–4)	0.63
Combination ATB treatment, n (%)	21 (95.4)	12 (92.3)	10 (100)	1.00
ATB treatment duration, median (IQR)	8 (7–14)	**7 (7–8)**	**12 (10–26)**	**0.03**
Corticosteroids on admission, n (%)	5 (22.7)	2 (15.4)	3 (30)	0.73
*Complications*				
Bacteremia, n (%)	10 (45.4)	9 (69.2)	1 (10)	**0.01**
ARDS, n (%)	6 (26.0)	4 (30.8)	2 (20)	0.91
Empyema, n (%)	4 (17.4)	2 (15.4)	2 (20)	1.00
Abscess, n (%)	1 (4.5)	0	1 (10)	0.89
Ventilatory support				
Invasive MV, n (%)	2 (8.5)	1 (7.7)	1 (10)	1.00
NIV, n (%)	2 (8.5)	0	2 (20)	0.34
HFNO, n (%)	12 (52.2)	10 (76.9)	2 (20)	**0.02**
*Clinical resolution time*				
Highest/lowest leukocytosis, median (IQR), days	5.5 (3–10)	3 (2–6)	6 (5–9)	0.06
Highest/lowest fever, median (IQR) days	0.5 (0–1)	**0.3 (0–1)**	**1 (0–1.5)**	**0.03**
Highest/lowest secretions, median (IQR) days	3 (2–5)	2 (2–4)	3.5 (1.5–5)	0.47
Highest/lowest infiltrates, median (IQR) days	9 (5–12)	**7 (5–11)**	**11 (9–20)**	**0.02**
Highest/lowest PaO2/FiO2, median (IQR) days	3 (2–5)	3 (2–6)	3 (2–4)	0.90
*Outcomes*				
Crude mortality in ICU, n (%)	2 (8.7)	2 (15.4)	0	0.58
Crude mortality in Hospital, n (%)	2 (8.7)	2 (15.4)	0	0.58
ICU stay (days), median (IQR)	5.5 (3–10)	5 (3–8)	6 (4–10)	0.70
Hospital stay (days), median (IQR)	13.5 (9–20)	13 (10–17)	15 (9–26)	0.53

Abbreviations: Acute physiology and chronic health evaluation (APACHE) II score; confusion, uremia, respiratory Rate, BP, and age ≥ 65 years (CURB-65); clinical pulmonary infection score (CPIS); sequential organ failure assessment (SOFA); intensive care unit (ICU); mechanical ventilation (MV); noninvasive ventilation (NIV); high-flow nasal oxygen therapy (HFNO); acute respiratory distress syndrome (ARDS); antibiotic (ATB). * A total of 4 episodes *Staphylococcus aureus* (1 of which is *Methicillin-resistant Staphylococcus aureus*, 2 episodes of *Haemophilus influenzae,* 3 episodes of *Legionella pneumophila*, 1 episode of *Streptococcus pyogenes*).

**Table 2:** Biomarker levels at day 1 regarding pneumococcal and non-pneumococcal sCAP.

	Overall (n = 23)	MyD	Pn (n = 13)	MyD	Non-Pn (n = 10)	MyD	*p*
Tn							
Day 1 (median, IQR)	19.3 (14–27)	1 (4%)	22.0 (14–27)	1 (7.7%)	14.0 (14.23)	0	0.25
Day 3 (median, IQR)	13.8 (13–14)	0	14.0 (13.6–13.9)	0	14.0 (17.8–14.4)	0	0.06
day 7 (median, IQR)	39 (15–61)	11 (48%)	49 (18–58)	7 (54%)	30 (14–63)	4 (40%)	0.45
h-FABP							
Day 1 (median, IQR)	2.4 (1.5–4.3)	2 (8.7%)	2.3 (1.4–3.1)	0	2.4 (1.9–4.7)	2 (20%)	0.43
Day 3 (median, IQR)	2.4 (1.2–3.6)	1 (4.4%)	2.6 (0.9–3.8)	1 (7.7%)	2.0 (1.3–3.3)	1 (10%)	0.92
Day 7 (median, IQR)	1.3 (0.9–1.5)	1 (4.4%)	1.4 (1.0–1.6)	0	1.0 (0.6–1.4)	1 (10%)	0.25
NT-proBNP							
Day 1 (median, IQR)	1800 (1570–2033)	20 (87%)	1730 (1090–1860)	10 (77%)	1848 (1690–2166)	10 (100%)	0.33
Day 3 (median, IQR)	1424 (1100–1695)	18 (78%)	1370 (967–1600)	10 (77%)	1500 (1300–2265)	8 (80%)	0.43
Day 7 (median, IQR)	1170 (941–1455)	17 (74%)	1250 (1040–1500)	10 (77%)	1150 (898–1295)	7 (70%)	0.87

Abbreviations: Myocardial damage (MyD); pneumococcal (Pn); ultrasensitive Troponin T (Tn); heart fatty acid binding protein (H-FABP); N-terminal pro-brain natriuretic peptide (NT-proBNP).

**Table 3:** Biomarker levels at day 1, 3, 7 regarding pneumococcal and non-pneumococcal sCAP.

	Overall (n = 23)	PN (n = 13)	Non-Pn (n = 10)	*p*-Value
*IL-1*				
Day 1 (median, IQR)	48.8 (35.9–53.4)	48.8 (43.2–54.0)	45.3 (33.8–52.0)	1.0
Day 3 (median, IQR)	44.1 (20.9–53.1)	46.3 (35.5–51.2)	36.7 (16.4–72.6)	0.9
Day 7 (median, IQR)	19.9 (16.5–21.0)	19.8 (17.3–21.6)	16.8 (9.7–19.0)	0.2
*Histone*				
Day 1 (median, IQR)	0.26 (0.23–0.28)	0.27 (0.23–0.29)	0.24 (0.23–0.27)	0.41
Day 3 (median, IQR)	0.28 (0.26–0.30)	0.28 (0.27–0.30)	0.27 (0.24–0.30)	0.43
Day 7 (median, IQR)	0.46 (0.38–0.52)	0.48 (0.45–0.53)	0.34 (0.23–0.47)	0.05
DNAs				
Day 1 (median, IQR)	37.3 (33.2–45.7)	37.3 (35.7–45.4)	38.6 (33.1–45.8)	0.87
Day 3 (median, IQR)	37.4 (34.7–40.5)	38.2 (35.5–42.8)	35.5 (33.5–38.2)	0.16
Day 7 (median, IQR)	33.7 (33.0–35.0)	33.8 (33.4–35.3)	33.6 (29.4–34.5)	0.40

**Table 4:** Cardiac injury and cell injury biomarker levels at day 1, 3, 7 regarding survival status.

	Alive (n = 21)			Dead (n = 2)		
	Day 1	Day 3	Day 7	Day 1	Day 3	Day 7
Tn (median, IQR)	19 (14–27)	171 (14–471)	38 (14–64)	21 (18–24)	571 (500–640)	55 (53–57)
h-FABP (median, IQR)	2.2 (1.4–3.1)	2.2 (1.2–3.0)	1.1 (0.9–1.5) *	4.7 (4.5–4.9)	3.9 (3.8–3.9)	2.0 (1.8–2.2) *
Histones (median, IQR)	0.25 (0.23–0.27)	0.27 (0.26–0.30)	0.47 (0.38–0.53)	0.28 (0.28–0.29)	0.30 (0.30–0.30)	0.45 (0.44–0.46)
IL-1 (median, IQR)	48 (31–50)	36 (19–54)	17 (16–20)	54 (54–54)	49 (48–51)	23 (22–24)
DNA (median, IQR)	37 (33–44)	37 (34–39)	33 (32–34)	47 (46–48)	47 (46–47)	36 (35–36)

* *p* = 0.04. Abbreviations: Ultrasensitive Troponin T (Tn); heart fatty acid binding protein (H-FABP).

## Data Availability

Data are contained within the article and Supplementary Materials.

## Supplementary Materials

The following supporting information can be downloaded at: https://www.mdpi.com/article/10.3390/antibiotics12121710/s1, Figure S1: Power calculation analysis.
