# Supplementary figures and images for: A Multicentric Observational Study to Determine Myocardial Injury in Severe Community-Acquired Pneumonia (sCAP)
Source: Antibiotics (Basel). 2023 Dec 8;12(12):1710. doi: 10.3390/antibiotics12121710 (PMC10740668; doi:10.3390/antibiotics12121710)

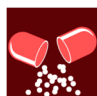

Supplementary material.

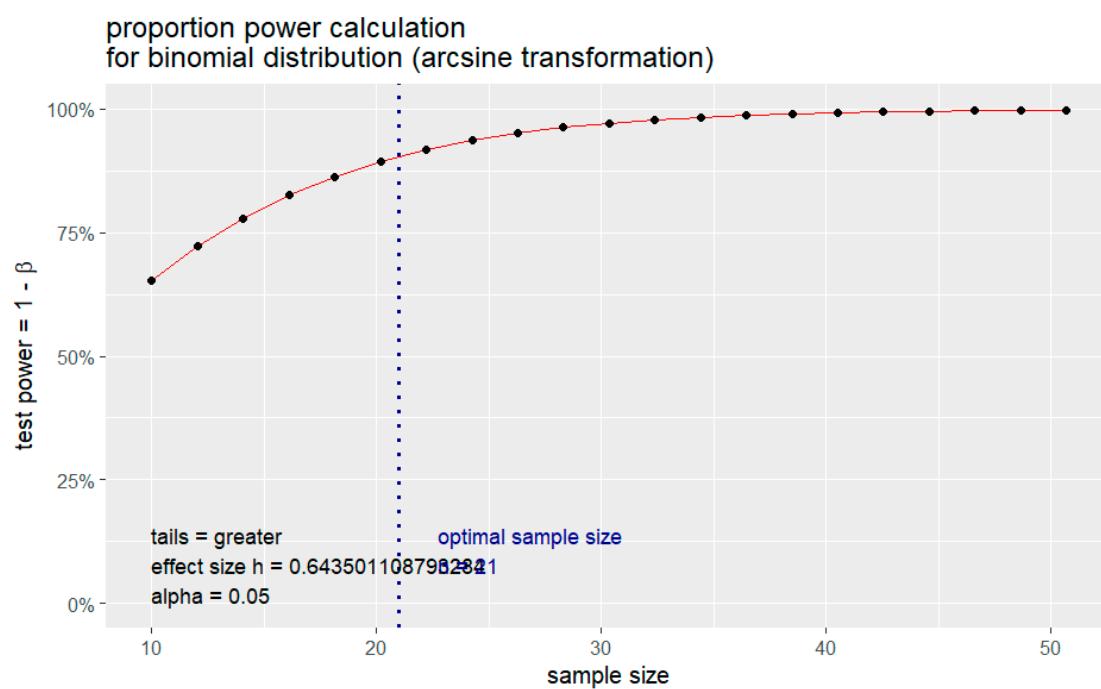

Figure S1. Power calculation analysis.

Supplement: Supplementary file 1 [file antibiotics-12-01710-s001.zip › antibiotics-2725731-supplementary.pdf]
